# Assessing and adjusting for non-response in the Millennium Cohort Family Study

**DOI:** 10.1186/s12874-017-0294-8

**Published:** 2017-01-28

**Authors:** Nida H. Corry, Christianna S. Williams, Mike Battaglia, Hope Seib McMaster, Valerie A. Stander

**Affiliations:** 1 Abt Associates Inc., Central Park West, Suite 210, 5001 South Miami Boulevard, 27703 Durham, NC USA; 2 Military Population Health Directorate at the Naval Health Research Center, 140 Sylvester Road, 92106 San Diego, CA USA; 3 Henry M. Jackson Foundation for the Advancement of Military Medicine, 6720A Rockledge Drive, Suite 100, 20817 Bethesda, MD USA

**Keywords:** Nonresponse bias, US military, Dyadic recruitment, Propensity score modeling, Survey weighting

## Abstract

**Background:**

In conducting population-based surveys, it is important to thoroughly examine and adjust for potential non-response bias to improve the representativeness of the sample prior to conducting analyses of the data and reporting findings. This paper examines factors contributing to second stage survey non-response during the baseline data collection for the Millennium Cohort Family Study, a large longitudinal study of US service members and their spouses from all branches of the military.

**Methods:**

Multivariate logistic regression analysis was used to develop a comprehensive response propensity model.

**Results:**

Results showed the majority of service member sociodemographic, military, and administrative variables were significantly associated with non-response, along with various health behaviours, mental health indices, and financial and social issues. However, effects were quite small for many factors, with a few demographic and survey administrative variables accounting for the most substantial variance.

**Conclusions:**

The Millennium Cohort Family Study was impacted by a number of non-response factors that commonly affect survey research. In particular, recruitment of young, male, and minority populations, as well as junior ranking personnel, was challenging. Despite this, our results suggest the success of representative population sampling can be effectively augmented through targeted oversampling and recruitment, as well as a comprehensive survey weighting strategy.

## Background

Military families and communities serve as the cornerstone of support for US service members and may themselves be heavily impacted by military experiences including deployment [1–6]. The US Administration, the Department of Defense, and the Institute of Medicine have identified military family research as a national priority, encouraging studies that examine the unique risk and resilience factors among military families and assess the resources they need in order to promote familial adjustment and optimal health outcomes [7–9]. While the literature in this area has progressed substantially in the last decade [10–12], there is much to learn about the functioning of military families, the influence of family interactions on service member recovery and resiliency, and the relationship of military experiences, such as deployment with family well-being. Population-based survey research is uniquely positioned to address these critical research questions and inform policy decisions to support service members and their families; however, very few longitudinal studies have been conducted with representative samples of military families. In recent years, several large-scale studies have been implemented among active duty military families and spouses from all US service branches including the Deployment Life Study [13], the Millennium Cohort Family Study (Family Study) [14], the Intergenerational Impact of War Study [15], the Military Family Life Project [16, 17], and the Survey of Active-Duty Spouses [18]. However, several of these studies excluded important subgroups (e.g., dual military, reserve couples) which limits the generalizability of their findings, and only three (Deployment Life Study, Military Family Life Project, and Family Study) are positioned to address questions regarding the trajectory of experiences for military families due to their longitudinal design.

The Family Study, a dyadic longitudinal survey of married spouses and military members with two to five years of service, addresses several of these gaps. The study aims to examine the well-being of military spouses and children in a probability-based longitudinal sample of service members from all branches and components of the military. As a result, it provides a unique empirical resource for addressing critical scientific, operational, and policy questions, and for informing the development of interventions promoting resilience among service members and their families. In addition to its robust design as a prospective, longitudinal survey of a probability-based sample across military branches, the Family Study addresses important gaps in the literature by including under-studied subsamples, such as Reserve and National Guard families, dual military couples, and male spouses. The study further plans to follow families over 21 years, a much longer period of time than ever previously attempted. Only married couples of opposite sex were included in the study; thus results may not generalize to lesbian, gay, bisexual and transgendered spouses or to single-parent households. The Family Study is an adjunct to the Millennium Cohort Study, which is the first US population-based prospective study to investigate long-term health effects of military service among active duty military. The Millennium Cohort Study was launched in 2001 and has enrolled four panels of service members; the first wave of enrollment for military spouses in the Family Study occurred concurrently with recruitment for the fourth panel (Panel 4; 2011–2013). The Family Study baseline employed a nested design in which spouses of service members who completed the Millennium Cohort survey were subsequently invited to participate in the study.

Unit non-response is an inevitable feature of population-based surveys, and it is important to thoroughly examine and adjust for potential non-response bias to improve the representativeness of the sample prior to conducting analyses of the data and reporting findings [19, 20]. The literature on non-response in population-based surveys indicates the following sociodemographic characteristics are often associated with greater survey response in the civilian population: employment [21, 22], middle versus younger age [21, 23], female gender [23], higher socioeconomic status/income [24, 25], and higher levels of formal education [26, 27]. Surveys of the military population generally reveal a similar pattern of greater response associated with these sociodemographic variables [28–30], as well as military-specific variables, such as active versus reserve duty status, officer versus enlisted status, and senior versus junior pay grade/rank [13, 27]. However, very little is known about the response patterns/behaviors of military spouses and family members. Although several spousal survey studies adjust for non-response [13, 16, 31], very few have provided any detailed discussion on the factors associated with spousal non-response. Findings suggest that a higher percentage of response is likely from officer-headed households across services [13]. In longitudinal surveys, participation in early data collection waves and fewer missing items are also associated with future participation [22, 32]; therefore, study participation is a predictor of future response. Less is known regarding the influence of psychosocial factors on non-response in the military population, as researchers typically do not have such measures on non-responders or on a proxy for the non-responder (e.g., family member). Some authors, however, suggest that non-respondents may generally be less healthy [25, 33, 34], have more substance use disorders [25, 34], or a history of psychiatric conditions [27].

This current study examined factors contributing to second stage survey non-response during the baseline data collection for the Millennium Cohort Family Study conducted from 2011 to 2013. Due to its nested design within the larger Millennium Cohort Study, the Family Study offers a unique opportunity to thoroughly examine and adjust for non-response bias among military spouses by analysing extensive data collected from their service member partners on the Millennium Cohort survey, including sociodemographic and psychosocial characteristics. This study contributes to the interpretation and use of the Family Study data by describing the sample and examining and addressing systematic non-response in the baseline sample. The study provides insights to inform future study designs and recruitment practices involving military spouses.

## Methods

### Study design

This study was an examination of second stage non-response bias to the baseline wave of a population-based, longitudinal survey of spouses of US active duty military conducted in 2012. We also present weighted population estimates of the sociodemographic characteristics of survey respondents, accounting for both sampling design and two stages of dyadic non-response.

### Study population, data, and procedures

Married spouses of participants in the Millennium Cohort Panel 4 were invited to participate in the study. This panel included only military members with two to five years of service randomly sampled across service branches and components. Female and married service members were oversampled to ensure adequate representation in the Family Study, particularly male spouses of female service members. Response rates for the Millennium Cohort have been described elsewhere (Williams C, Battaglia MB, Corry NH, McMaster H, Stander V. Millennium Cohort Family Study Weighting Analyses Overview document, 2016). Following completion of the Panel 4 survey, married service members were given the option to refer their spouse to the Family Study and provide their contact information, although this was not a requirement for spousal participation. Spouses who were referred by the service member were recruited via email and postal mail, while non-referred spouses were contacted by postal mail only. In order to successfully engage spouses, additional enrollment strategies included systematic variation in the style of recruitment solicitations, minimal ($5–$10) pre- and post-incentives, as well as both online and paper mail survey response options. The Family Study methods are described in more detail elsewhere [14, 35].

For the 28,603 service members who completed the Millennium Cohort survey and were eligible for the Family Study survey, we had extensive self-report data on which to model spousal non-response. Therefore, we utilized both administrative and service member self-report data to assess non-response bias among spouses. The study was overseen and approved by the Naval Health Research Center’s Institutional Review Board (Protocol 2000.0007) and the Office of Management and Budget (approval number 0720–0029). Informed consent was obtained for all participants.

### Measures

#### Survey administration

Service members' study participation status was determined by the percentage of “base” items completed on the Millennium Cohort survey (i.e., items that were asked of all participants); a participant was designated as a completer if >80% of the base items had responses and as a partial completer if ≤80% of the base items had responses. All Millennium Cohort respondents were categorized into one of two recruitment groups: those who referred their spouse for the Family Study and those who either refused to refer their spouse or submitted the survey without responding to the referral item.

#### Sociodemographic data

Sociodemographic and military data were obtained from service member military records and included gender, birth date, race/ethnicity, education, branch of service, service component, military pay grade, military occupation, deployment, and number of dependents.

#### Mental, physical, and social well-being

The Millennium Cohort survey solicited service member self-report data on a variety of topics, including medical conditions, psychosocial well-being, substance use, and military-specific and occupational exposures. Major depression was assessed by the Patient Health Questionnaire (PHQ) [36] and mental and physical health component scores were derived from the Veterans RAND 36 Item Health Survey (VR-36) [37, 38]. Stressful life events were assessed by the modified Holmes and Rahe scale [39, 40]. Posttraumatic stress disorder (PTSD) was assessed by the PTSD Checklist-Civilian Version [41]. Risk and resilience indicators were also assessed, including posttraumatic growth, from a modified Post Traumatic Growth Inventory [42], and self-mastery, by items from the Pearlin–Schooler Mastery Scale [43]. Health conditions and behaviours were assessed by the Insomnia Severity Index [44], CAGE questionnaire [45], and selected items from the National Health and Nutrition Examination Survey [46] and the National Health Interview Survey [47].

The Family Study survey consisted of approximately 100 items spanning spousal physical and mental health, reports on children’s adjustment, and family functioning. Many of the measures in the spousal survey were identical to those administered in the Millennium Cohort service member survey, so that outcomes could be compared and examined for the spousal dyad. More information about the Family Study survey instruments are available elsewhere [14]. For most of the analyses presented here, the key measure was simply whether or not the spouse completed the Family Study survey (i.e., responded to at least one survey item).

### Statistical analyses

#### Modeling family study non-response

We conducted bivariate analyses including Chi-Square tests and bivariate logistic regressions to identify key service member characteristics associated with spousal response. Subsequently, we used multivariate logistic regression analysis to develop a comprehensive response propensity model. Because methodological and background variables were most strongly associated with non-response and are more generally available in studies of non-response, the first multivariate logistic regression model included all sociodemographic, military, and administrative variables. In the second model, other service member characteristics that were bivariately associated with non-response were offered for stepwise addition (*P* < 0.05) to the first model. In order to include all Family Study eligible Millennium Cohort respondents (*N* = 28,603) in modeling non-response, missing cases were assigned the modal response for items with a very small amount of missing data (e.g., education). For other items, a “missing” category was created as one of the response levels for the analyses.

#### Testing the non-response models

We used several techniques to evaluate the model, since one of the purposes for developing a comprehensive model of spousal non-response was to adjust the survey weights for non-response bias. First, we computed the area under the receiver operating characteristic curve (C-statistic), which provided an estimate of the overall model fit. We also compared the distribution of the predicted probabilities of response derived from the models to ensure there was a broad distribution of response propensity. Second, in developing the propensity model, we held out several key service member variables that were strongly correlated with important Family Study outcomes; these variables could then be used as reasonable proxies in validating the effectiveness of the model to adjust for response bias in important outcomes [48]. The ideal response propensity model is predictive of non-response and key survey outcome variables (i.e., the model can account for non-response bias in important outcomes). Finally, to ensure our non-response model was comprehensive, we assessed whether adding the previously held out service member variables to the phase 2 model would have contributed substantively to its predictive power.

#### Developing family study weights

In developing survey weights, we first accounted for the Millennium Cohort design features and non-response bias because the Family Study was nested within the Millennium Cohort Study. We created sample design weights for the stratified (i.e., by gender and marital status) sampling frame (*n* = 250,000) to generalize to the population of military personnel with two to five years of service (*n* = 573,437). We then adjusted the sample design weights for Millennium Cohort non-response using raking ratio estimation [49] so the marginal totals of the adjusted weights matched those for the population [50]. The available service member data for this raking process was limited to demographic and service characteristics documented in military records and included gender, age, race/ethnicity, pay grade, service branch, and military component (active duty vs. Reserve/National Guard). The Millennium Cohort weight (MilCo weight) could be applied to analyses involving Panel 4 participants eligible for the Family Study (*N* = 28,603) and was used for all non-response analyses presented in this paper.

In the next stage of Family Study weight development, we used the estimated spousal response propensities derived from the logistic regression model described above to directly adjust the weights, generating weights adjusted for both Millennium Cohort and Family Study non-response [51]. As a final step, we raked the weights again to known population totals for gender, race/ethnicity, age, pay grade, and service branch, as well as the family study response propensity quintile and trimmed them to reduce weight variability without sacrificing the approximation to those population totals [52]. The resulting weight can be applied to statistical analysis of the 9872 dyads that participated in the Family Study. We evaluated the effectiveness of the weights in reducing non-response bias by comparing the prevalence of sociodemographic characteristics estimated in three ways: unweighted, applying the MilCo weight, and applying the final Family Study weight.

## Results

### Baseline characteristics and study participation

A total of 9872 spouses responded to the Family Study survey, out of 28,603 eligible service member respondents, for an overall response rate of 34.5% (34.3% using the MilCo weight). For all eligible Millennium Cohort service members, Table 1 presents the prevalence of each of the demographic, administrative, military, and individual adjustment characteristics included in our non-response model, as well as percentages of spouses responding to the Family Study survey by subgroup. Table 2 further lists Millennium Cohort participant characteristics that were found to have a statistically significant relationship with spousal response in bivariate analyses, but were not included in the final model.

**Table 1:** Characteristics of Millennium Cohort participants and Family Survey response – included in final response propensity model

Characteristic	Prevalence of characteristics^a^		Percent responding to Family Survey	*P*-value
	Unweighted *n*	Prevalence		
Total	28,603	100.00	34.3	
Demographic, service, and administrative characteristics				
Gender				
Male	21,984	86.0	37.1	<0.0001
Female	6619	14.0	16.6	
Age				
17–24 years	12,378	48.3	31.0	<0.0001
25–29 years	10,797	35.5	37.5	
30+ years	5428	16.2	37.1	
Race/ethnicity				
White, non-Hispanic	21,294	69.6	37.6	<0.0001
Black, non-Hispanic	2470	12.1	22.1	
Hispanic	2602	10.8	29.1	
Other	2237	7.5	30.2	
Education				
< High school completion/diploma	51	0.2	19.9	<0.0001
High school degree/GED/or equivalent	5477	24.2	29.8	
Some college (including 2 years degree)	15,469	57.4	33.4	
Bachelor’s degree	5414	14.1	41.9	
Advanced degree	2192	4.1	46.7	
Income				
< $50,000	15,912	62.5	33.5	<0.0001
$50,000–$74,999	5799	18.4	37.2	
$75,000+	4817	11.1	41.1	
Missing	2075	7.9	24.2	
Number of dependent children				
None	12,664	44.6	32.2	<0.0001
One	7865	27.5	35.2	
Two	5205	18.0	36.6	
Three or more	2869	9.8	36.8	
Component				
Active duty	22,365	78.9	34.1	0.1408
Reserve/National Guard	6238	21.1	35.1	
Service branch				
Army	12,949	50.3	34.7	0.0001
Navy/Coast Guard	4698	17.1	35.7	
Marine Corps	2649	15.3	34.4	
Air Force	8307	17.4	31.6	
Pay grade				
Enlisted	23,505	91.0	32.8	<0.0001
Warrant/commissioned officer	5098	9.0	48.7	
Deployment/combat				
Not deployed	6276	19.5	31.3	<0.0001
Deployed, no combat	3357	10.7	33.7	
Deployed, in combat	17,977	65.9	36.0	
Deployed, combat unknown	993	4.0	21.4	
Panel 4 survey completion				
Complete	27,045	93.9	35.1	<0.0001
Partial	1558	6.1	20.8	
Panel 4 respondent referred spouse				
Yes	8319	28.9	64.5	<0.0001
No	20,284	71.1	22.0	
Health behaviours				
Average hours of sleep/day				
<6 h	6848	27.76	32.2	<0.0001
6 h	8192	28.95	34.1	
7 h	6939	21.53	39.2	
8 h	4707	15.01	34.1	
>8 h	1298	4.43	32.4	
Missing	619	2.33	19.7	
Description of usual activities				
Sit during day and do not walk much	6581	20.5	35.2	<0.0001
Stand/ walk a lot, little lifting	11,052	37.5	32.9	
Light loads, or climb often	7893	29.4	35.5	
Heavy work	2499	10.3	36.8	
Missing	578	2.2	20.5	
Physical health indices				
Body mass index				
<25.0	10,612	34.3	33.7	<0.0001
25.0–29.9	13,710	49.5	35.3	
30.0+	3893	14.9	33.9	
Missing	388	1.3	14.7	
Days unable to work due to illness/injury				
None	16,622	58.2	34.4	<0.0001
1–5 days	6456	21.1	34.6	
6–15 days	2196	7.9	32.9	
16+ days	2657	10.4	35.7	
Missing	672	2.4	25.0	
Mental health indices				
VR-36 mental component score				
Lowest 15%	3611	14.1	31.6	<0.0001
Middle 70%	19,279	66.5	35.4	
Highest 15%	4335	14.0	35.9	
Missing	1378	5.3	22.6	
Having no one to turn to when you have a problem				
Not bothered	22,717	77.4	35.6	<0.0001
Bothered a little	3699	14.0	31.2	
Bothered a lot	1674	6.8	29.8	
Missing	513	1.8	18.5	
Financial problems or worries				
Not bothered	16,001	51.7	35.0	<0.0001
Bothered a little	8846	33.1	35.4	
Bothered a lot	3314	13.6	30.7	
Missing	442	1.6	18.2	
Overall attitude about military service				
Negative/somewhat negative	4773	17.3	32.8	<0.0001
Neither negative or positive	3713	13.7	30.7	
Positive/somewhat positive	18,264	61.8	36.7	
Missing	1853	7.12	23.4	
Trauma				
Stressful events since 2008				
None	12,035	40.9	34.8	<0.0001
1 or 2	11,434	40.0	36.2	
3 or more	3626	13.3	32.6	
Missing	1508	5.9	21.1	

^a^Unless specified as "unweighted", all table values are weighted with the Normalized Adjusted Weight 1 [Design weight with raking adjustment for non-response to P4W1]

**Table 2:** Measures examined for association with Family Study response – not included in final response propensity model

Service Member Characteristic^a^	
Health behaviours	
Daily number of caffeinated beverages	
Physical health indices	
VR-36 physical component score^b^	
Insomnia severity	
Mental health indices	
Major depression disorder^b^	
PTSD symptoms using the specific criteria^b^	
Posttraumatic growth	
Self-mastery	
Substance use	
Potential alcohol dependence^b^	
Smoking status^b^	
Treatment	
Days hospitalized because of illness or injury	
Stress and relationship issues	
Difficulties with spouse or partner^b^	
Little or no sexual desire/pleasure during sex	
Stress at work, outside the home, or at school	
Trauma	
Physically hurt or unwanted sexual act	

^a^Significant at the *p* < 0.0001 level

^b^Indicates measures that were held out of stepwise selection process for purposes of model validation (see text for further description)

Note: We examined several additional variables, but they did not remain significant in the non-response propensity model and were somewhat duplicative of variables presented here, thus were not included in the table. These variables included: Panel 4 questionnaire mode; number of daily sedentary hours; anxiety; PTSD; alcohol-related problems; current tobacco use (not cigarettes); use of psychiatric medication; history of complementary and alternative medicine treatment; stress of taking care of family; number of lifetime stressful events

The majority of service member sociodemographic, military, and administrative variables were significantly associated with greater spousal response, including male gender, older age, white/non-Hispanic ethnicity, higher education, an income between $25,000–$74,999, having dependent children, serving in the Reserve/National Guard versus active duty, having been deployed with known combat status, completing the full Millennium Cohort survey, and referring spouse to the Family Study. Service member health behaviours such as smoking, low or high sleep duration (vs. intermediate), greater caffeine intake, and sedentary activities, as well as poorer overall physical health indices, were associated with lower survey response. Positive mental health indices were associated with greater likelihood of spousal response, including the absence of major depression, PTSD symptoms, medication, and more posttraumatic growth. Fewer financial and social problems, including fewer difficulties with partner and stressful life events, were also related to greater spousal response.

Although many of these comparisons were statistically significant, the most substantive differences were observed for a fairly small number of demographic and administrative variables. For example, the single strongest predictor of spousal response was whether the service member referred him/her for participation, with 64.5% of referred spouses participating compared to only 22% of non-referred spouses. Other factors associated with at least a 50% difference in spousal response from Table 1 included gender, with spouses of male service members more than twice as likely to respond as spouses of female service members (37.1% vs. 16.6%); race/ethnicity (with spouses of minority service members much less likely to respond than those of white, non-Hispanic service members); education (with spousal response rates increasing steadily from 19.9% for service members not completing high school to 46.7% for those with an advanced degree); and income (with those in middle income groups much more likely to respond than those in the highest income group). Notably, for several service member measures, spousal response was considerably lower for those with missing data on the given measure; there was less variation among the non-missing categories. Further, missingness was positively correlated across variables, as evidenced by the much greater spousal response for service members completing the entire survey (35.1%) compared with partial completers (20.8%).

### Family Study non-response models

Table 3 presents results from the weighted logistic regression models. In the first model, only the service member sociodemographic, military, and administrative variables were included. The second model shows other characteristics that were selected in the subsequent stepwise regression because they were significantly associated with response, above and beyond the variance accounted for by the sociodemographic, administrative, and military variables. These included physical health indices (i.e., amount of sleep, activity level, body mass index, work days missed due to illness or injury), overall mental health, social isolation, financial and social distress, attitude toward military service, and number of recent stressful life events. As in the unadjusted analyses, spouse referral (adjusted odds ratio (AOR) 6.53, 95% confidence interval (CI) 6.16–6.93), female versus male service member (AOR 0.33, 95% CI 0.31–0.37); and minority race/ethnicity (AOR 0.55, 95% CI 0.50–0.61 for black compared with white non-Hispanic service members) were the strongest correlates of spousal response.

**Table 3:** Multiple logistic regression for participation in the Millennium Cohort Family Study

Service member characteristic	Model 1: demographic, military and administrative characteristics		Model 2: all characteristics	
	Odds ratio (95% CI)	*P*-value	Odds ratio 95% CI)	*P*-value
Demographic, service, and administrative characteristics				
Gender				
Female	0.32 (0.30–0.36)	<0.0001	0.33 (0.31–0.37)	<0.0001
Male	Reference		Reference	
Age group				
17–24 years	0.93 (0.85–1.02)	0.0305	0.93 (0.84–1.01)	0.0215
25–29 years	1.02 (0.94–1.11)		1.01 (0.93–1.10)	
30+ years	Reference		Reference	
Race/ethnicity				
Black, non-Hispanic	0.54 (0.49–0.60)	<0.0001	0.55 (0.50–0.61)	<0.0001
Hispanic	0.76 (0.69–0.83)		0.77 (0.70–0.84)	
Other	0.76 (0.68–0.84)		0.77 (0.70–0.86)	
White, non-Hispanic	Reference		Reference	
Education				
< High school completion/diploma	0.59 (0.30–1.14)	<0.0001	0.83 (0.42–1.65)	<0.0001
High school graduate/GED	0.83 (0.77–0.88)		0.83 (0.78–0.90)	
Advanced degree	1.32 (1.12–1.56)		1.30 (1.10–1.54)	
Bachelor's degree	1.22 (1.10–1.35)		1.20 (1.08–1.32)	
Some college	Reference		Reference	
Income				
< $50,000	0.88 (0.79–0.98)	<0.0001	0.91 (0.82–1.02)	0.0411
$50,000–$74,999	0.96 (0.86–1.07)		0.97 (0.87–1.08)	
Missing	1.24 (1.03–1.50)		1.20 (0.93–1.55)	
$75,000+	Reference		Reference	
Number of dependent children				
Three or more	0.99 (0.90–1.10)	0.0121	1.01 (0.91–1.12)	0.0043
Two	1.10 (1.02–1.19)		1.12 (1.03–1.21)	
One	1.09 (1.02–1.17)		1.11 (1.03–1.18)	
None	Reference		Reference	
Component				
Reserve/National Guard	1.04 (0.97–1.12)	0.2587	1.03 (0.96–1.11)	0.3963
Active duty	Reference		Reference	
Service branch				
Air Force	0.98 (0.90–1.06)	0.0104	0.92 (0.84–1.00)	0.0008
Marine Corps	1.05 (0.97–1.14)		1.05 (0.97–1.14)	
Navy/Coast Guard	1.13 (1.04–1.23)		1.11 (1.02–1.21)	
Army	Reference		Reference	
Paygrade				
Officer	1.38 (1.20–1.57)	<0.0001	1.35 (1.18–1.54)	<0.0001
Enlisted	Reference		Reference	
Deployment/combat				
Deployed, in combat	1.05 (0.98–1.14)	0.5210	1.09 (1.01–1.18)	0.1490
Deployed, no combat	1.07 (0.96–1.19)		1.07 (0.96–1.19)	
Deployed, unknown combat	0.97 (0.76–1.24)		0.93 (0.71–1.22)	
Not deployed	Reference		Reference	
P4 interview completion				
Partial	0.61 (0.48–0.77)	<0.0001	0.64 (0.42–0.96)	0.0296
Complete	Reference		Reference	
P4 respondent referred spouse				
Yes	6.41 (6.04–6.79)	<0.0001	6.53 (6.16–6.93)	<0.0001
No	Reference		Reference	
Health behaviours				
Average hours of sleep/day				
<6 h			0.84 (0.77–0.93)	<0.0001
6 h			0.91 (0.83–1.00)	
7 h			1.08 (0.99–1.18)	
>8 h			1.10 (0.94–1.28)	
Missing			0.92 (0.69–1.23)	
8 h			Reference	
Description of usual activities				
Heavy work			1.12 (1.00–1.24)	0.0066
Light loads, or climb often			1.04 (0.96–1.13)	
Stand/walk a lot, little lifting			0.95 (0.88–1.02)	
Missing			1.11 (0.82–1.50)	
Sit during day, walk little			Reference	
Body mass index				
30.0+			0.98 (0.90–1.07)	0.0120
25.0–29.9			0.97 (0.91–1.04)	
Missing			0.57 (0.41–0.80)	
< 25.0			Reference	
Days work missed due to illness or injury				
16+ days			1.19 (1.08–1.31)	0.0124
6–15 days			1.01 (0.91–1.13)	
1–5 days			1.03 (0.96–1.10)	
Missing			1.07 (0.86–1.32)	
None			Reference	
Mental health				
VR-36 mental component				
Highest 15%			0.97 (0.90–1.06)	0.0405
Lowest 15%			0.99 (0.90–1.09)	
Missing			1.41 (1.10–1.81)	
Middle 70%			Reference	
Stress and relationship issues				
Stress: no one to turn to				
Bothered a lot			0.92 (0.80–1.05)	0.0085
Bothered a little			0.89 (0.81–0.97)	
Missing			0.65 (0.45–0.95)	
Not bothered			Reference	
Stress: financial				
Bothered a lot			0.81 (0.73–0.89)	0.0007
Bothered a little			0.95 (0.89–1.01)	
Missing			0.88 (0.57–1.36)	
Not bothered			Reference	
Attitude towards military				
Negative/somewhat negative			0.92 (0.85–1.00)	0.0007
Neither negative or positive			0.85 (0.78–0.92)	
Missing			1.07 (0.79–1.43)	
Positive/somewhat positive			Reference	
Stressful events since 2008				
3 or more			1.06 (0.97–1.17)	0.0347
1 or 2			1.09 (1.03–1.16)	
Missing			0.84 (0.54–1.32)	
None			Reference	

### Model testing and calculation of final family study weights

The C-statistics for both models (0.764 and 0.768, respectively) indicated acceptable discrimination [53]. The overall mean predicted probability of response for the models was 0.343. For both models, the mean predicted probability of response was markedly higher for responders (0.482–0.485) than for non-responders (0.269–0.270). To choose the optimal non-response model from which to derive the response propensity adjustment for the Family Study weights, we estimated logistic regression models. For these models, we used the same set of independent variables as the two non-response models for each of the selected primary service member measures withheld from the response propensity models (i.e., PHQ potential alcohol dependence, current smoker, former smoker, PHQ major depression, VR-36 physical component, PTSD symptoms using specific criteria, and difficulties with partner). These measures were chosen because they paralleled family member measures which were considered a key outcome for the Family Study, and the service member measure (proxy) was at least modestly correlated with the corresponding family member measure (correlations ranged from 0.109 to 0.492 among responders). For most of the dependent variables, there was a marked improvement in fit between Model 1 and Model 2 as evaluated by changes in the C-statistic, which ranged from 0.629 to 0.958. In particular, for major depression, poor physical health, PTSD symptoms, and partner difficulties, the C-statistic improved by more than 20%. For example, for major depression, the C-statistics for models 1 and 2 were 0.743 and 0.958 respectively. There was little improvement in fit for smoking status (for current smoking, the C-statistic went from 0.730 to 0.746 between Model 1 and Model 2 and for former smoking the C-statistics were 0.629 and 0.634 respectively). Based on these analyses and the higher C-statistic for Model 2, we selected this model to estimate response propensity for adjusting the Family Study weights. Adding the withheld service member outcome measures did not substantively improve the model fit.

The MilCo weights were directly adjusted using the predicted probabilities from Model 2. Next, we again applied raking ratio estimation to rebalance the weights of the population totals. As a final step, the weights were trimmed to eliminate extreme weights and mitigate the variance inflation associated with applying the weights. The trimming of the weights reduced the largest weight by 41% (270.2 to 159.4) and increased the lowest weight by 153% (1.75 to 4.44). After the raking and trimming, weighted estimates for all control margins were still very close to population estimates. As expected the coefficient of variation (CV) of the weights does increase with each successive adjustment, reflecting the increase in variance that is often associated with non-response bias reduction. For Family Study responders, the CV was 0.197 for the design weight, 0.487 for the weight adjusted for MilCo non-response, and 0.973 for the final weight adjusted for Family Study non-response.

### Sociodemographic estimates and impact of weights

Table 4 describes sociodemographic characteristics of the Family Study respondents estimated in three ways: unweighted, applying the MilCo weight, and applying the final Family Study weight. We also showed the population estimates derived from military records for the entire sampling frame.

**Table 4:** Weighted and unweighted estimates for Family Study respondent characteristics (*n* = 9872)

Characteristic	*n*	Unweighted	Weighted for service member non-response (MilCo weight) % (SE)	Weighted with final Family Study weight % (SE)	Population estimates^a^
Service member characteristics					
Gender					
Male	8599	87.1	93.2 (0.25)	86.0 (0.35)	86.0
Female	1273	12.9	6.8 (0.25)	14.0 (0.35)	14.0
Race					
White	7956	80.6	76.4 (0.43)	69.6 (0.46)	69.6
Black	522	5.3	7.8 (0.27)	12.1 (0.33)	12.1
Other	1394	14.1	15.7 (0.37)	18.2 (0.39)	18.2
Age					
17–24 years	3676	37.2	43.7 (0.50)	48.3 (0.50)	48.3
25–34 years	5364	54.3	51.1 (0.50)	46.8 (0.50)	46.8
35+ years	832	8.4	5.3 (0.23)	4.9 (0.22)	4.9
Paygrade					
Enlisted	7456	75.5	87.2 (0.34)	91.0 (0.29)	91.0
Warrant/commissioned officer	2416	24.5	12.8 (0.34)	9.0 (0.29)	9.0
Service branch					
Army	4562	46.2	50.9 (0.50)	50.3 (0.50)	50.3
Navy/Coast Guard	1683	17.0	17.8 (0.39)	17.1 (0.38)	17.1
Marine Corps	932	9.4	15.3 (0.36)	15.3 (0.36)	15.3
Air Force	2695	27.3	16.0 (0.37)	17.4 (0.38)	17.4
Component					
Active duty	7685	77.8	78.4 (0.42)	78.9 (0.41)	78.9
Reserve/National Guard	2187	22.2	21.6 (0.42)	21.1 (0.41)	21.1
Deployment/combat					
Not deployed	1999	20.2	17.8 (0.39)	19.1 (0.40)	
Deployed, no combat	1122	11.4	10.5 (0.31)	10.7 (0.31)	
Deployed, in combat	6530	66.1	69.2 (0.47)	65.9 (0.48)	
Deployed, combat unknown	221	2.2	2.5 (0. 16)	4.3 (0.20)	
Number of dependent children					
None	4097	41.5	41.8 (0.50)	42.8 (0.50)	
One	2788	28.2	28.3 (0.46)	28.3 (0.45)	
Two	1912	19.4	19.3 (0.40)	18.7 (0.39)	
3+	1075	10.9	10.6 (0.31)	10.2 (0.30)	
Education					
< High school completion/diploma	10	0.1	0.1 (0.04)	0.2 (0.04)	
High school graduate/GED	1583	16.0	21.0 (0.41)	24.3 (0.43)	
Some college	4929	49.9	56.1 (0.50)	56.7 (0.50)	
Bachelor's degree	2318	23.5	17.2 (0.38)	14.8 (0.36)	
Advanced degree	1032	10.5	5.6 (0.23)	4.1 (0.20)	
Income					
< $50,000	5203	52.7	61.1 (0.49)	62.0 (0.49)	
$50,000–$74,999	2170	22.0	20.0 (0.40)	18.6 (0.39)	
$75,000+	1996	20.2	13.3 (0.34)	11.1 (0.32)	
Unknown	503	5.1	5.6 (0.23)	8.2 (0.28)	
Spouse characteristics					
Gender					
Male	1274	12.9	6.9 (0.26)	14.0 (0.35)	
Female	8598	87.1	93.1 (0.26)	86.0 (0.35)	
Age					
17–24 years	4001	40.5	47.5 (0.50)	49.0 (0.50)	
25–34 years	4836	49.0	44.5 (0.50)	43.0 (0.50)	
35+ years	877	8.9	6.5 (0.25)	6.6 (0.25)	
Unknown	158	1.6	1.5 (0.12)	1.3 (0.12)	
Race					
White	7678	77.8	75.0 (0.44)	70.5 (0.46)	
Black	411	4.2	5.5 (0.23)	8.1 (0.27)	
Other	1717	17.4	18.9 (0.40)	20.7 (0.41)	
Unknown	66	0.7	0.6 (0.08)	0.7 (0.08)	
Education					
< High school completion/diploma	133	1.3	1.6 (0.13)	1.7 (0.13)	
High school graduate/GED	1145	11.6	13.1 (0.34)	14.7 (0.36)	
Some college	4569	46.3	51.1 (0.50)	53.0 (0.50)	
Bachelor's degree	2841	28.8	25.3 (0.44)	23.0 (0.42)	
Advanced degree	1162	11.8	8.7 (0.28)	7.3 (0.26)	
Unknown	22	0.2	0.2 (0.05)	0.2 (0.05)	
Income					
< $50,000	5324	53.9	63.1 (0.49)	64.2 (0.48)	
$50,000–$74,999	2321	23.5	21.3 (0.41)	21.1 (0.41)	
$75,000+	2091	21.2	14.3 (0.35)	13.0 (0.34)	
Unknown	136	1.4	1.4 (0.12)	1.6 (0.13)	
Military service					
Current	916	9.3	6.7 (0.25)	9.4 (0.29)	
Former	840	8.5	7.7 (0.27)	10.1 (0.30	
Never	8107	82.1	85.5 (0.36)	80.5 (0.40)	
Unknown	9	0.1	0.1 (0.03)	0.1 (0.03)	

^a^Target population estimates used as raking margins; these estimates are not available for other characteristics

The Family Study sample included mostly white females, over 90% of whom were under the age of 34. The majority of spouses were partnered with enlisted personnel versus warrant/commissioned officers (91.1 and 9.0%, respectively), and the most commonly represented branches were the Army (50.8%), Air Force (17.4%), and Navy (17.1%). Most spouses were married to an active duty service member (78.9%), and the vast majority had experienced at least one combat-related deployment separation (80.9%) at time of the survey. Nearly 20% of spouses were currently serving or had served in the military.

The difference between unweighted estimates and those weighted with the MilCo weight showed the effect of stratified sampling and Millennium Cohort non-response, while the comparison of unweighted data and those weighted with the Family Study weights showed the combined effects of study design and both stages of non-response. In all cases, final weighted estimates closely mirrored the population estimates which showed the weighting achieved the intended result. For some measures, the combined effects of sampling design and non-response led to the unweighted estimates which were very close to the final weighted estimates (as well as to the population); this was particularly true for gender. Although gender was associated with response at both stages, the combination of non-response bias, along with the oversampling of female service members, resulted in unweighted estimates being very close to those in the targeted population. Similarly, the prevalence of spouses of active duty and Reserve/National Guard service members in the sample was quite similar to their representation in the population. Differences were more striking for age, race/ethnicity, and pay grade, where the unweighted estimates differed quite substantially from the weighted and population values. For example, non-Hispanic blacks were present in the Family Study sample at less than half the prevalence in the population, while those aged 35 and older were nearly twice as prevalent in the sample as in the population. For service branch and component, the effects were intermediate. The standard errors of the estimates generally did not increase dramatically with the adjustment for second-stage non-response.

## Discussion

This study examined correlates of second stage non-response at time of baseline data collection for the Millennium Cohort Family Study in order to provide important context for future study results and to establish a foundation for non-response adjustment. A total of 9,872 spouses participated in the Family Study for an overall response rate of 34.5% (34.3% using the MilCo weight). The probability sample of military spouses included spouses of Reserve/National Guard and dual-military couples, as well as an oversampling of male spouses of service members, all of whom are often under-represented subgroups in military family research. Consistent with the objectives of the Family Study, the majority of couples had experienced at least one combat-deployed separation.

The results of our initial unadjusted non-response analyses showed the majority of service member sociodemographic, military, and administrative variables were significantly associated with greater spousal response, along with various health behaviours, mental health indices, and financial and social issues. Similar to other survey research studies, we found that sociodemographic characteristics and survey administrative factors were most strongly associated with response propensity. In multivariate analyses, the most important correlates of spousal response were service member gender, age, race/ethnicity, education, income, number of dependent children, service branch, and Millennium Cohort completion status. The single strongest predictor of spousal response was if the spouse was referred by the service member for participation. Finally, as is commonly the case in health survey research studies, we found that Family Study respondents were slightly better adjusted, with fewer physical and mental health symptoms, than non-respondents. However, effects associated with health and well-being were small.

The Family Study developed weights to adjust for non-response using a propensity modeling and raking strategy. In support of this, one of the important objectives of this analysis was to choose the optimal logistic regression model from which to derive the response propensity adjustment. We made this selection by applying two alternative models to predict several key service member measures that had been withheld from the models for purposes of validation. For most of these dependent variables, there was a substantial improvement in fit between Model 1 (sociodemographic/administrative variables only) and Model 2 (sociodemographic/administrative, physical, mental health, and stressful life event variables); thus Model 2 was ultimately selected. This ensured variables in the response propensity model fit the two critical criteria for reducing non-response bias-association, both with the likelihood of response and study outcomes of greatest interest. It is also important to note that adding these reserved study outcomes to the response propensity model as a final check did not appreciably improve the fit.

Differential non-response occurs in a military survey when the response rate varies for sample subgroups, typically defined by demographic and service member characteristics. This can often be corrected by making non-response adjustments. However, non-response adjustments can only account for factors that have been measured in administrative records or in the course of the study, and most spousal surveys have limited information on the dyad to assess non-response. The Family Study is uniquely positioned to offer insights into spousal response because there is a wealth of information from the service member to utilize for both spousal responders and non-responders, including physical and mental health indices. It is important to note tha﻿t despite the inclusion of a multitude of variables in the non-response models in the current study, sociodemographic factors alone performed nearly as well in predicting non-response. Further, although the breadth of variables examined in this study exceeds the typically narrow set of socio-demographic characteristics used to assess non-response in military studies, additional military-related variables could be of interest in future studies such as whether the family lives on base and time on duty station.

Our results suggest future studies should combine targeted oversampling and comprehensive response bias adjustment strategies to address the problem of non-response in population subgroups that are difficult to engage, and should assess sociodemographic factors to help examine and adjust for non-response. For example, since male spouses are a group known to be difficult to recruit, the Millennium Cohort Panel 4 study design oversampled married and female service members in order to better facilitate Family Study enrollment. Due to this oversampling, the final population of male spouses was almost the same as their proportion in the actual population of young spouses despite a substantially lower response rate among males. Ultimately, the combination of oversampling and the application of comprehensive non-response adjustment techniques used in the Family Study provided the opportunity to represent the subgroup of military couples involving a female service member and male spouse more comprehensively than any previous research effort. This type of combined approach would be helpful in future military family research, not only with male spouses, but also spouses of service members who are enlisted, younger (17–24 years old), and black or Hispanic personnel, all of whom are less likely to respond and may require additional or enhanced recruitment strategies.

In addition to oversampling, the Family Study designed a range of targeted recruitment materials to engage participants who may be difficult to enroll. A good match between survey topic and participant characteristics has been found to influence response in prior research [54]; therefore, our study team was concerned with the difficulty in recruiting spouses who did not identify strongly with the “military spouse” or “military family” community. Once again, we were particularly concerned that male spouses may not have a strong military spouse self-schema; dual-military partners also may not identify with this role. The team had further concerns that couples without children may not strongly identify with the “military family schema”. Despite inclusion of a broad definition of military families in study recruitment materials, results indicated some spouses in the sampling frame may have been influenced by a more narrowly defined military family schema in making the choice of whether or not to respond. Furthermore, an examination of the Family Study service member referral process suggested Millennium Cohort participants may have been influenced by similarly narrow military family schemas in deciding whether to encourage their marital partners to engage in the Family Study (McMaster H, Stander V, O’Malley C, Williams C, Woodall K, Bauer L; unpublished observations, manuscript in development). Unfortunately, Millennium Cohort participants were given minimal information regarding the breadth of targeted participation for the Family Study in their introduction to the project. Given that referral was a strong correlate of response likelihood in this study, future dyadic research investigations may improve subgroup representation and overall participant response by enhancing both partners’ understanding of the intended recruitment population. Future military family research should consider that some military spouses may not feel as well integrated into the military community as others, and this may influence response rates.

## Conclusions

In summary, our results indicated the Family Study was significantly impacted by a number of non-response factors that commonly affect survey research. In particular, recruiting young, male, and minority populations as well as junior ranking personnel was challenging. Despite this, the Family Study successfully employed multiple strategies to minimize the impact of non-response bias, including a comprehensive propensity modeling approach to develop weights. Our results suggest the success of representative population sampling can be effectively augmented through targeted oversampling and recruitment, as well as more comprehensive survey weighting strategies. In military populations where more extensive information is available documenting characteristics of the total population, future studies could take advantage of available data in adjusting for non-response bias.

Ultimately, the Family Study enrolled a uniquely large dyadic cohort with extensive self-report and military archival data available for all 9872 couples. This is a substantial representation from subgroups that are difficult to engage and frequently excluded in military family research (e.g., male spouses, reservists, dual-military couples). A particular goal was to engage a study panel of junior military personnel and their families. These relatively new members of the military community are an at-risk group, and this young cohort could be followed over the course of their military careers and beyond, capturing critical life events such as divorce as well as separation from the military and associated outcomes. Further, the Family Study participants entered the military community at a time when they likely would be maximally impacted by operations in Afghanistan and Iraq. As such, this program of research presents a critical opportunity to understand the impact of deployment and military life stress on family well-being. This study demonstrates that by conducting comprehensive non-response bias analyses, accounting for a myriad of constructs potentially associated with non-response, and applying weights to adjust for those factors, the data are much more representative of the target population. Although the methodology is not novel in and of itself, this study clearly demonstrates the benefits of non-response modeling and weighting in bias minimization. Ultimately, these weighted data provide the opportunity to generalize to military spouses whose partners had two to five years of military experience as of 2011.

Currently, the study team is exploring deployment-related stressors, as well as mediating factors, influencing outcomes, such as spousal depression, substance use, and marital satisfaction. In future work, we plan to address a full spectrum of issues related to spouse and child physical and mental health, as well as marital and family adjustment. This study will be able to investigate areas that previous military family research has never examined, such as long-term outcomes for families impacted by deployment separations, and longitudinal trajectories of family adjustment over the course of career military service and beyond.

## Abbreviations

AOR: Adjusted odds ratio
CI: Confidence interval
C-statistic: Area under the receiver operating characteristic curve
Family Study: The department of defense millennium cohort family study
MilCo weight: Millennium cohort study weight
PHQ: Patient health questionnaire
PTSD: Posttraumatic stress disorder
VR-36: Veterans RAND 36 item health survey
